# Incidence, Risk Factors, and Modified Risk Assessment Model of Venous Thromboembolism in Non‐Hodgkin Lymphoma Patients

**DOI:** 10.1002/cam4.70510

**Published:** 2024-12-23

**Authors:** Wen Li, Rui Liu, Ying Shen, GongZhizi Gao, Rui Yang, Yiwen Wang, Ruoyu Yang, Zujie Lin, Ruijun Dong, Wanhong Zhao, Aili He, Ju Bai

**Affiliations:** ^1^ Department of Hematology The Second Affiliated Hospital of Xi'an Jiaotong University Xi'an Shaanxi China; ^2^ Department of Clinical Laboratory The Second Affiliated Hospital of Xi'an Jiaotong University Xi'an Shaanxi China; ^3^ Xi'an Key Laboratory of Hematological Diseases Xi'an China; ^4^ National‐Local Joint Engineering Research Center of Biodiagnostics and Biotherapy The Second Affiliated Hospital of Xi'an Jiaotong University Xi'an Shaanxi China; ^5^ Department of Tumor and Immunology, Precision Medical Institute Xi'an Jiaotong University Xi'an China

**Keywords:** non‐Hodgkin lymphoma, risk assessment model, ThroLy score, thrombin–antithrombin complex, thrombomodulin, venous thromboembolic event

## Abstract

**Background:**

Venous thromboembolic events (VTEs) are the second‐leading cause of death in cancer patients, with an incidence of 5%–17% in lymphoma patients, particularly higher in those with non‐Hodgkin lymphoma (NHL). Existing risk assessment models (RAMs) like the Khorana and ThroLy scores have limitations and are inadequately validated for NHL patients. Coagulation markers such as D‐dimer, thrombin–antithrombin complex (TAT), and thrombomodulin (TM) show a potential predictive value for cancer‐associated VTE but lack extensive research in NHL.

**Objectives:**

This study aimed to analyze characteristics and predictive risk factors for VTE in newly diagnosed NHL patients and to evaluate and improve the clinical applicability of the Khorana and ThroLy scores.

**Methods:**

Data were collected from newly diagnosed NHL patients to analyze characteristics and potential predictive risk factors for VTE. The clinical applicability of the Khorana and ThroLy scores was evaluated, and the ThroLy score was improved by adjusting the hemoglobin cutoff and combining it with D‐dimer testing. Sequential testing with TM and TAT levels was also performed.

**Results:**

VTE developed in 7.09% of NHL patients. Independent risk factors for VTE included clinical Stage III/IV, mediastinal involvement, history of VTE, D‐dimer≥ 1345 μg/dL, and platelet (PLT)≥ 298 × 10^9^, and Hb≥ 110 g/L was an independent protective factor for VTE. The ThroLy score was improved by adjusting the hemoglobin cutoff and combining it with D‐dimer testing. Sequential testing of TM and TAT achieved a sensitivity of 66.7%, specificity of 100%, positive predictive value (PPV) of 100%, and negative predictive value (NPV) of 96.7%.

**Conclusions:**

VTE is a significant complication in NHL patients. The study highlighted independent risk factors and proposed a modified risk assessment model that effectively predicted VTE risk, potentially optimizing prevention and reducing healthcare costs.

**Abbreviations**
APTT: activated partial thromboplastin time
AUC: the area under the ROC curve
CI: confidence interval
D‐D: D‐dimer
ECOG: Eastern Cooperative Oncology Group
FDP: fibrinogen degradation product
FIB: fibrinogen
HB: hemoglobin
HL: Hodgkin lymphoma
INR: international normalized ratio
LDH: lactate dehydrogenase
MPV: mean platelet volume
NE: neutrophil count
NHL: non‐Hodgkin lymphoma
NLR: neutrophil‐to‐lymphocyte ratio
NPV: negative predictive value
OR: odds ratio
PDW: platelet distribution width
PIC: plasmin–‐antiplasmin complex
PICC: peripherally inserted central catheter
PLT: platelet
PPV: positive predictive value
PT: prothrombin time
RAM: risk assessment models
RBC: red blood cell
RDW‐SD: red blood cell distribution width
TAT: thrombin–‐antithrombin complex
TM: thrombomodulin
t‐PAIC: tissue plasminogen activator/plasminogen activator inhibitor‐1 complex
TT: thrombin time
VTE: venous thromboembolic event
WBC: white blood cell

## 1 Introduction

Thrombosis is a serious and common complication in cancer patients, with the risk being 3–4 times higher in cancer patients compared to the general population [1, 2]. Venous thromboembolic event (VTE) is the second‐leading cause of death in cancer patients, often leading to poor prognosis [3]. The incidence rate of VTE is closely associated with cancer types, with gastrointestinal cancer, lung cancer, and brain tumors having particularly high rates of VTE [4]. Notably, recent study has shown that the incidence of VTE in hematologic malignancies, especially in lymphoma, is comparable to or even higher than that in solid tumors [5]. The reported incidence rate of VTE in lymphoma patients ranged from 5% to 17% [6, 7, 8, 9], with non‐Hodgkin lymphoma (NHL) patients showing a higher incidence than those with Hodgkin lymphoma (HL) [10, 11].

Assessing VTE risk and implementing preventive strategies for high‐risk patients is crucial in cancer therapy [12]. Several risk assessment models (RAMs) have been established to evaluate the risk for VTE. Caprini score, developed in 1991, is recommended to apply in surgery patients [13, 14, 15]. The Khorana score is derived from a multicenter research on chemotherapy‐related VTE in cancer patients [16], while it includes various types of cancers, with lymphoma only being a part of the cohort. In addition, the efficiency of the Khorana score in evaluating VTE in lymphoma patients is controversial, with some studies validating its efficacy [17], while others reported a low positive predictive value for lymphoma‐associated VTE [18]. The ThroLy score has been established to specifically evaluate venous thrombosis risk in lymphoma patients, including NHL, HL, and chronic lymphocytic leukemia/small lymphocytic lymphoma, theoretically providing a more accurate assessment [19]. However, it also has limitations, such as small patient samples from only two clinical centers and insufficient external validation. Rupa‐Matysek [20] evaluated the association of ThroLy with VTE in patients treated for diffuse large B‐cell lymphoma (DLBCL) or HL undergoing first‐line chemotherapy; however, the performance of THroLy score was not adequately accurate possibly due to differences in patient populations. Therefore, further research is needed to assess the predictive performance of these scores in lymphoma patients and to explore new indicators to enhance the diagnostic efficiency of the scoring system.

The pathogenesis of VTE in NHL has not been not fully understood. Certain coagulation markers, such as D‐dimer, thrombin–antithrombin complex (TAT), tissue plasminogen activator/plasminogen activator inhibitor‐1 complex (t‐PAIC), thrombomodulin (TM), and plasmin‐antiplasmin complex (PIC), have been extensively studied for their predictive value in cancer‐associated VTE [21]. TM, a type I transmembrane protein, is mainly expressed in endothelial cells and could indicate the function of endothelia cells, and TAT levels could reflect the coagulation system activation. PIC levels indicate fibrinolytic system activation, and t‐PAIC is crucial in regulating fibrinolysis and directly influences thrombus formation and degradation. Zhou et al. [22] found the predictive value of TAT, PIC, TM, and t‐PAIC for cancer‐associated VTE. However, there is limited research on these markers in NHL patients.

In this study, we collected data from newly diagnosed NHL patients treated at the Second Affiliated Hospital of Xi'an Jiaotong University, analyzed the characteristics and potentially predictive risk factors of VTE, and evaluated the clinical applicability of the Khorana and ThroLy scores. In addition, we improved the diagnostic efficiency of ThroLy score by modifying the cutoff value of hemoglobin and combining with D‐dimer in simultaneous testing. Upon the combinative testing, we found that TM and TAT levels were significantly increased in high‐risk patients and further sequential testing of TM + TAT could remarkably enhance the diagnostic efficacy. The modified risk assessment model offered a more effective prediction method for VTE risk in NHL patients, potentially optimizing VTE prevention and reducing healthcare costs.

## 2 Materials and Methods

### 2.1 Patient Selection

We retrospectively collected data from January 2016 to December 2019 on newly diagnosed NHL patients who sought initial treatment at the Department of Hematology, Second Affiliated Hospital of Xi'an JiaoTong University. All cases met the inclusion criteria: (1) newly diagnosed patients; (2) pathological diagnosis of all NHL patients was based on the WHO/Lugano criteria [23]; and (3) all NHL patients underwent at least one cycle of chemotherapy. (4) Venous thromboembolism was identified as deep vein thrombosis, pulmonary embolism, or both, which was diagnosed based on established criteria and supported by imaging data, including color Doppler ultrasound as the gold standard for deep vein thrombosis, pulmonary angiography, and spiral CT pulmonary angiography for pulmonary embolism [24]. Exclusion criteria are as follows: (1) patients who refused treatment or did not undergo chemotherapy; (2) patients with incomplete medical records during follow‐up; (3) patients with concomitant malignancies; and (4) patients on long‐term medication affecting coagulation function.

Newly diagnosed B‐cell NHL patients in this study received the R‐CHOP regimen, which includes rituximab, cyclophosphamide, doxorubicin, vincristine, and prednisone. For T‐cell lymphoma, patients were treated with either the CHOP regimen combined with etoposide (CHOP‐E) or DA‐EPOCH. In cases of NK/T‐cell lymphoma, the P‐GemOx regimen or radiotherapy was administered.

For the validation cohort, we collected newly diagnosed NHL patients at the Department of Hematology, Second Affiliated Hospital of Xi'an JiaoTong University, from January 2020 to June 2021 according to the criteria above. To collect venous blood from patients, 1.8 mL of blood will be drawn into sodium citrate anticoagulant tubes with a concentration of 0.109 mmol/L. The tubes will then be centrifuged at 1500 × *g* for 10 min to separate platelet‐poor plasma, which will be stored at −80°C for future testing.

### 2.2 Data Collection

We collected (1) general characteristics, including age, gender, body mass index (BMI), smoking status, alcohol consumption, medical history, history of blood transfusion, presence of comorbidities, history of thrombosis, and surgical history within 6 months prior to diagnosis; (2) clinical features, including NHL pathological type, clinical stage, Eastern Cooperative Oncology Group (ECOG) score, presence of B symptoms, and use of peripherally inserted central catheter (PICC), mediastinal involvement, time and location of VTE formation; and (3) hematological parameters at the initial diagnosis before chemotherapy, including white blood cell (WBC) count, neutrophil count (NE), neutrophil‐to‐lymphocyte ratio (NLR), red blood cell (RBC) count, hemoglobin (HB), platelet (PLT) count, red blood cell distribution width (RDW‐SD), mean platelet volume (MPV), platelet distribution width (PDW), D‐dimer (D‐D), international normalized ratio (INR), lactate dehydrogenase (LDH), fibrinogen (FIB), activated partial thromboplastin time (APTT), prothrombin time (PT), thrombin time (TT), and fibrinogen degradation product (FDP).

### 2.3 Assessment of VTE Risk

In this study, we conducted both Khorana score [16] and ThroLy score [19] to evaluate the risk of VTE in patients with NHL. The detailed score systems are shown in Tables S1 and S2. For the Khorana score, high risk is defined as ≥ 3, intermediate risk as 1–2, and low risk as 0. Similarly, for the ThroLy score, high risk is > 3, intermediate risk is 2–3, and low risk is 0–1. To improve the diagnostic efficiency of present risk model, we performed simultaneous and sequential testing. In simultaneous diagnostic testing, multiple tests were administered at the same time; a patient only needs to test positive on one of the tests to be considered positive. In sequential diagnostic testing, tests were administered one after another based on the results of previous tests, and only those who consistently test positive across multiple tests are considered positive.

### 2.4 Statistical Analysis

Student *t*‐test was employed for continuous variables, while chi‐square test was used for categorical variables. Logistic regression was utilized to for multivariate analysis, identifying risk factors for VTE in NHL patients. The applicability of Khorana and ThroLy scores was compared using the area under the ROC curve (AUC). The optimal cutoff values for hematological parameters were determined based on ROC curves using sensitivity and specificity. Survival analysis was conducted using Kaplan–Meier method, with *p*‐value determined by the Log Rank test. *p* < 0.05 indicated statistical significance. The statistical analyses were performed using SPSS software (IBM Corporation, Armonk, NY, USA) and GraphPad Prism 8 for data visualization and additional statistical analysis.

## 3 Results

### 3.1 Patients' Characteristics

The retrospective cohort comprised 325 eligible NHL patients. General characteristics are presented in Table S3. Male cases constituted 51.7%, and patients over 60 years old accounted for 52.6%. The predominant NHL type was diffuse large B‐cell lymphoma, representing 48.9%. Other notable types included NK/T‐cell lymphoma (13.2%), peripheral T‐cell lymphoma (10.2%), and follicular lymphoma (5.4%). Ann Arbor Stage II exhibited the most frequent at 31.7%, followed by Stage III (23.7%), Stage IV (23.4%), and Stage I (21.2%).

In the cohort of 325 patients, as shown in Table 1, VTE occurred in 21 individuals (6.46%). The median time from diagnosis of lymphoma to VTE was 2.0 months. The time of VTE occurrence was predominantly within 3 months of chemotherapy (38.1%), followed by prior to chemotherapy initiation (28.6%). VTE formation sites varied, with upper limb venous thrombosis being the most prevalent (42.9%), followed by lower limb (28.6%). In addition, diffuse large B‐cell lymphoma was the most prevalent type associated with VTE (57.1%), followed by angioimmunoblastic T‐cell lymphoma (9.4%).

**TABLE 1:** Characteristics of VTE.

Characteristics	Case (%)
VTE	
VTE occurred	21 (6.46)
VTE not occurred	304 (93.54)
Time of VTE occurrence	
Before chemotherapy	6 (28.6)
≤ 3 months	8 (38.1)
3–6 months	4 (19.0)
> 6 months	3 (14.3)
Site of VTE formation	
Upper limb venous thrombosis	9 (42.9)
Lower limb venous thrombosis	6 (28.6)
Subclavian and jugular vein thrombosis	4 (19.0)
Left head vein thrombosis	2 (9.5)
Pathological NHL types of patients with VTE	
Diffuse large B‐cell lymphoma	12 (57.1)
Angioimmunoblastic T‐cell lymphoma	2 (9.4)
NK/T‐cell lymphoma	1 (4.8)
Peripheral T‐cell lymphoma	1 (4.8)
Mantle cell lymphoma	1 (4.8)
T‐cell prolymphocytic leukemia	1 (4.8)

**FIGURE 1:**
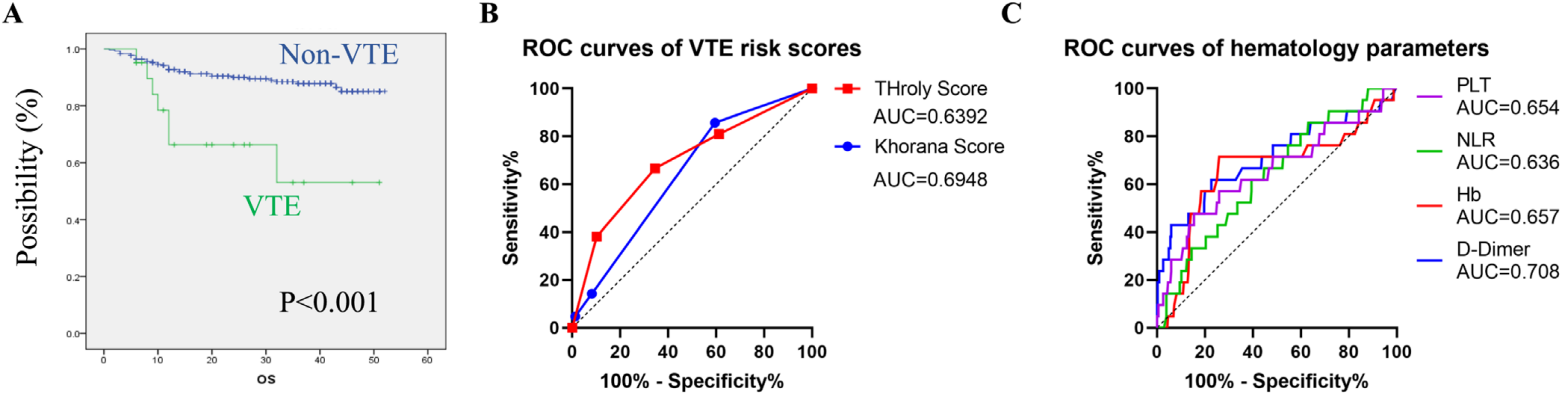
Assessment the diagnostic efficiency of ThroLy and Khorana scores. (A) Kaplan–Meier survival analysis of OS. (B) ROC curves of ThroLy and Khorana scores for VTE. (C) ROC curves of hematologic parameters for VTE. Hb, hemoglobin; NLR, neutrophil/lymphocyte ratio; OS, overall survival; PLT, platelet.

Among the 325 patients included in the study, 24 patients were lost to follow‐up and could not be contacted later. For the remaining 301 patients, the median follow‐up period was 26.5 months, and the median overall survival (OS) was not reached. We found that NHL patients developing VTE had significantly shorter OS compared to those without VTE (*p* < 0.001, Figure 1A). The 1‐year and 2‐year survival rates for NHL patients with VTE were 61.9% and 38.1%, respectively, compared to 87.4% and 66.8% for patients without VTE.

### 3.2 Risk Factors for VTE in NHL Patients

Univariate analysis confirmed that the following factors were associated with the development of VTE (Table 2): infection (*p* = 0.025), ECOG score ≥ 3 (*p* = 0.003), PICC placement (*p* = 0.049), clinical Stage III/IV (*p* = 0.001), mediastinal involvement (*p* < 0.001), and history of VTE (*p* < 0.001).

**TABLE 2:** Univariate analysis of risk factors for VTE in NHL patients.

Factors	Non‐VTE (*n* = 304) (%)	VTE (*n* = 21) (%)	*p*	OR	95% CI
Age					
≤ 60 years	143 (47.0)	11 (52.4)	0.635	0.807	0.333–1.957
> 60 years	161 (53.0)	10 (47.6)			
Gender					
Male	154 (50.7)	14 (66.7)	0.156	0.513	0.202–1.307
Female	150 (49.3)	7 (33.3)			
B symptoms					
Present	163 (53.6)	15 (71.4)	0.113	2.163	0.817–5.723
Absent	141 (46.4)	6 (28.6)			
BMI					
≥ 25 (kg/m^2^)	5 (1.6)	1 (4.8)	0.332	2.99	0.333–26.830
< 25 (kg/m^2^)	299 (98.4)	20 (95.2)			
Hypertension					
Present	47 (15.5)	2 (9.5)	0.752	0.576	0.130–2.554
Absent	257 (84.5)	19 (90.5)			
Diabetes					
Present	23 (7.6)	2 (9.5)	0.67	1.286	0.282–5.867
Absent	281 (92.4)	19 (90.5)			
Coronary heart disease					
Present	26 (8.6)	2 (9.5)	0.7	1.126	0.248–5.102
Absent	278 (91.4)	19 (90.5)			
Stroke history					
Present	27 (8.9)	3 (14.3)	0.426	1.71	0.473–6.178
Absent	277 (91.1)	18 (85.7)			
Traumatism history					
Present	40 (13.2)	2 (9.5)	1	0.695	0.156–3.096
Absent	264 (86.8)	19 (90.5)			
Smoking					
Present	79 (26.0)	5 (23.8)	0.826	0.89	0.316–2.509
None	225 (74.0)	16 (76.2)			
Alcohol use					
Yes	49 (16.1)	3 (14.3)	1	0.867	0.246–3.057
No	255 (83.9)	18 (85.7)			
EPO use					
Yes	32 (10.5)	2 (9.5)	1	0.895	0.199–4.019
No	272 (89.5)	19 (90.5)			
G‐CSF use					
Yes	39 (12.8)	3 (14.3)	0.742	1.132	0.319–4.023
No	265 (87.2)	18 (85.7)			
Blood transfusion					
Yes	29 (9.5)	3 (14.3)	0.447	1.58	0.439–5.689
No	275 (90.5)	18 (85.7)			
Infection					
Yes	42 (13.8)	7 (33.3)	**0.025**	3.119	1.189–8.179
No	262 (86.2)	14 (66.7)			
ECOG score					
≥ 3	45 (14.8)	9 (42.9)	**0.003**	4.317	1.720–10.836
< 3	259 (85.2)	12 (57.1)			
PICC placement					
Yes	72 (23.7)	9 (42.9)	**0.049**	2.417	0.979–5.967
No	232 (76.3)	12 (57.1)			
Clinical stage					
I/II	168 (55.3)	4 (19.0)	**0.001**	5.25	1.726–15.969
III/IV	136 (44.7)	17 (81.0)			
Mediastinal involvement					
Yes	41 (13.5)	10 (47.6)	**< 0.001**	5.831	2.330–14.595
No	263 (86.5)	11 (52.4)			
History of VTE					
Yes	4 (1.3)	6 (28.6)	**< 0.001**	30	7.644–117.742
No	300 (98.7)	15 (71.4)			

*Note:* Bold, statistical difference (*p* < 0.05).

Abbreviations: BMI, body mass index; CI, confidence interval; ECOG, eastern collaborative oncology group; EPO, erythropoietin; G‐CSF, granulocyte colony‐stimulating factor; OR, odds ratio; PICC, peripherally inserted central catheter; VTE, venous thromboembolic events.

In addition, we compared a panel of hematological parameters between NHL patients with and without VTE (Table 3). Patients with VTE displayed notably lower levels of HB in comparison to those without VTE (*p* = 0.024). Moreover, patients with VTE exhibited higher levels of NLR (*p* = 0.049), PLT (*p* = 0.039), and D‐Dimer (*p* = 0.012) compared to those without VTE. To incorporate these continuous variables into the multivariate logistic regression analysis, we calculated the cutoff values, and the cutoff values for D‐Dimer, Hb, PLT, and NLR were 1345 μg/dL, 110 g/L, 298 × 10^9^, and 2.11, respectively.

**TABLE 3:** Hematologic parameters of NHL patients with and without VTE.

Parameters	Non‐VTE	VTE	*p*
WBC (10^9^/L)	6.95 ± 4.57	7.55 ± 3.73	0.556
NE (10^9^/L)	6.55 ± 1.95	6.29 ± 1.98	0.418
NLR	3.33 ± 2.30	4.37 ± 2.45	**0.049**
RBC (10^12^/L)	4.06 ± 0.68	3.82 ± 0.88	0.244
HB (g/L)	120.92 ± 22.23	109.48 ± 24.47	**0.024**
PLT (10^9^/L)	205.49 ± 91.01	276.62 ± 145.84	**0.039**
MPV (fL)	11.11 ± 1.24	10.86 ± 1.42	0.375
PDW (fL)	13.50 ± 2.97	12.46 ± 2.97	0.123
RDW‐SD (fL)	46.94 ± 6.73	49.05 ± 7.52	0.168
LDH (u/L)	311.64 ± 280.28	271.43 ± 104.41	0.514
PT (s)	10.85 ± 1.32	10.30 ± 1.30	0.064
INR	0.96 ± 0.12	0.91 ± 0.09	0.077
APTT (s)	28.02 ± 7.21	25.13 ± 5.82	0.074
FIB (mg/L)	318.01 ± 105.05	284.95 ± 80.66	0.159
TT (s)	18.89 ± 1.87	18.45 ± 1.50	0.299
D‐dimer (ug/dL)	1171.15 ± 1025.55	3196.19 ± 3348.32	**0.012**
FDP (mg/L)	3.83 ± 4.21	7.93 ± 9.03	0.052

*Note:* Bold values indicate significant difference (*p* < 0.05).

Abbreviations: APTT, activated partial thromboplastin time; FDP, fibrinogen degradation product; FIB, fibrinogen; HB, hemoglobin; INR, International Normalized Ratio; LDH, lactate dehydrogenase; MPV, mean platelet volume; NE, neutrophilic granulocyte; NLR, neutrophil count to lymphocyte count ratio; PDW, platelet distribution width; PLT, platelet; PT, prothrombin time; RBC, red blood cell; RDW‐SD, red blood cell distribution width; TT, thrombin time; VTE, venous thromboembolic events; WBC, white blood cell.

Furthermore, we conducted multivariate logistic regression analysis based on the above factors (Table 4). Results showed that clinical Stage III/IV (*p* = 0.0013, OR = 7.523), mediastinal involvement (*p* = 0.001, OR = 11.274), history of VTE (*p* < 0.001, OR = 105.378), D‐dimer≥ 1345 μg/dL (*p* = 0.010, OR = 7.278), and PLT≥ 298 × 10^9^ (*p* = 0.016, OR = 6.110) were independent risk factors for VTE, and Hb≥ 110 g/L was an independent protective factor for VTE (*p* = 0.005, OR = 0.121).

**TABLE 4:** Multivariate logistic regression analysis of risk factors for VTE in NHL patients.

Risk factor	*B*	SE	Wald	*p*	OR
ECOG score ≥ 3	1.093	0.724	2.278	0.131	2.982
PICC placement	1.405	0.731	3.692	0.055	4.075
Clinical stage III/IV	2.018	0.812	6.182	**0.013**	7.523
Infection	0.860	0.765	1.262	0.261	2.363
Mediastinal involvement	2.423	0.754	10.309	**0.001**	11.274
History of VTE	4.658	1.238	14.157	**< 0.00** **1**	105.378
Hb ≥ 110 g/L	−2.112	0.755	7.822	**0.005**	0.121
D‐dimer ≥ 1345 μg/dL	1.985	0.768	6.684	**0.010**	7.278
PLT ≥ 298 × 10^9^	1.810	0.748	5.852	**0.016**	6.110
NLR ≥ 2.11	1.246	0.951	1.718	0.190	3.476

*Note:* Bold values indicate significant differences (*p* < 0.05).

Abbreviations: ECOG, Eastern Collaborative Oncology Group; OR, odds ratio; PICC, peripherally inserted central catheter; SE, standard error; VTE, venous thromboembolic events.

### 3.3 Assessing the Diagnostic Efficiency of Khorana Score and ThroLy Score

We assessed the diagnostic efficiency of Khorana score and ThroLy score by identifying high‐risk group as “positive” of diagnostic testing. Among all 325 patients, 28 were classified as high risk and 297 as intermediate risk according to the Khorana score, with 3 high‐risk patients developing VTE. The sensitivity, positive predictive value (PPV), specificity, negative predictive value (NPV), and Youden index of the Khorana score for predicting thrombosis in NHL patients were 14.3%, 10.7%, 91.8%, 93.9%, and 6.1, respectively. For the ThroLy score, 39 were classified as high risk, 164 as intermediate risk, and 122 as low risk, with 8, 8, and 5 patients developing VTE in each group, respectively. In addition, the ThroLy score demonstrated a sensitivity of 38.1%, PPV of 20.5%, specificity of 89.8%, NPV of 95.5%, and Youden index of 27.9. While the specificity of the ThroLy score was slightly lower than that of the Khorana score, all other predictive indices were higher for the ThroLy score. In addition, the AUC for the Khorana score was 0.639 (95% CI: 0.528–0.750, *p* = 0.033), while for the ThroLy score, it was 0.695 (95% CI: 0.568–0.821, *p* = 0.003) (Figure 1B). Taken together, the performance of ThroLy score was better than that of Khorana score in NHL patients of our cohort.

### 3.4 Improving the Diagnostic Performance of ThroLy Score by Combining D‐Dimer

As shown in Table 3, patients with VTE displayed lower levels of Hb and higher levels of NLR, PLT, and D‐dimer compared to those without VTE. Therefore, we first performed ROC analysis to investigate the diagnostic performance of these four parameters. Results showed that the AUC values for D‐dimer, Hb, PLT, and NLR were 0.708, 0.657, 0.654, and 0.636, respectively, suggesting that D‐dimer showed the highest diagnostic efficiency (Figure 1C).

**FIGURE 2:**
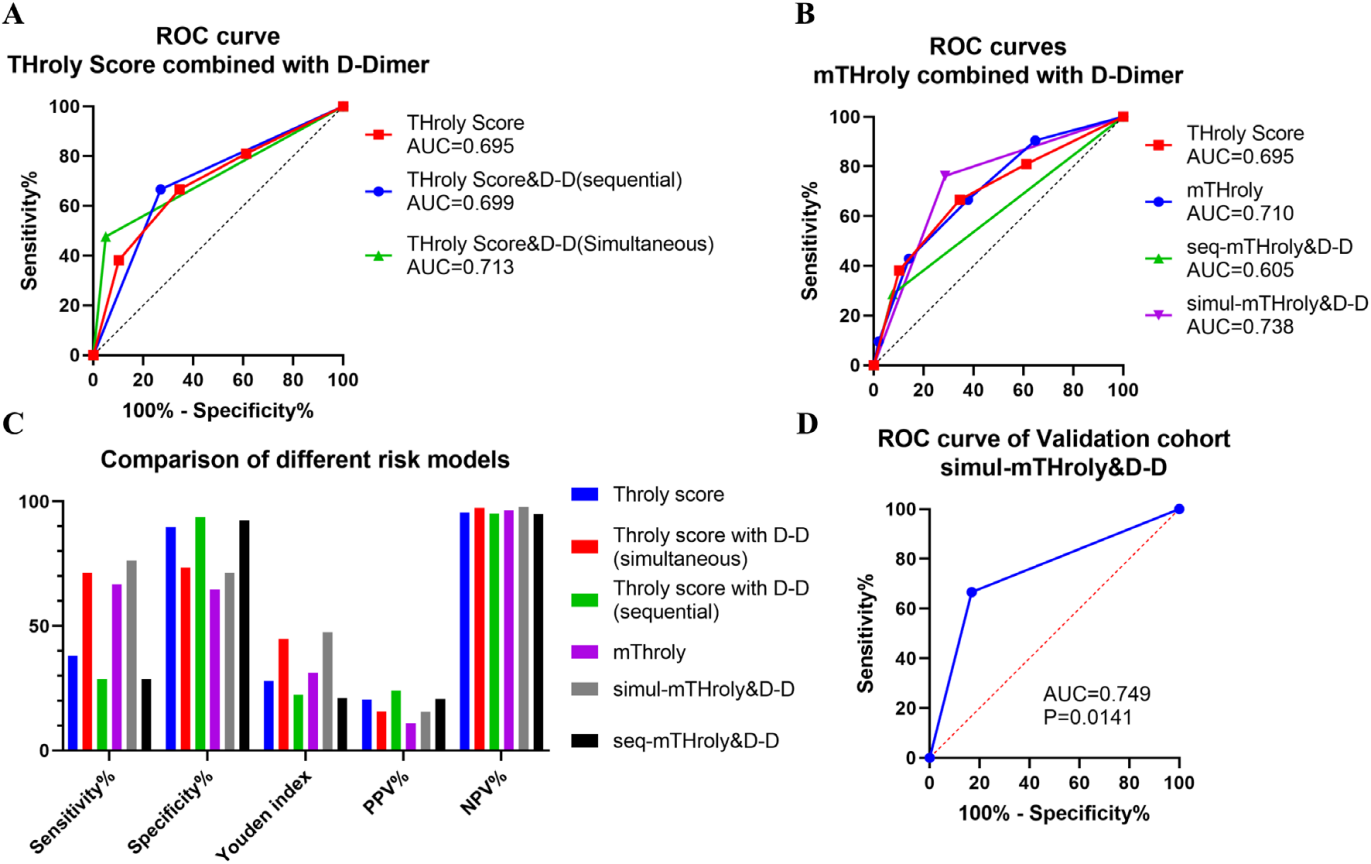
Improve the efficiency of ThroLy score and validate in the validation cohort. (A) ROC curves of ThroLy score combined D‐dimer in simultaneous (simul‐) and sequential (seq‐) testing for VTE. (B) ROC curves of modified ThroLy (mThroLy) score combined D‐dimer in simultaneous and sequential testing. (C) Comparison of the diagnostic efficiency of different risk models. (D) Validation of simul‐mThroLy&D‐D in the validation cohort.

To improve diagnostic performance, we combined ThroLy score with D‐dimer with the cutoff value of 1345 μg/dL in sequential testing and simultaneous testing. Results showed that the sequential testing could slightly enhance diagnostic efficacy, with the AUC value of 0.699, and simultaneous testing yielded an AUC value of 0.713 (Figure 2A).

Next, we adjusted the Hb cutoff of ThroLy score from 100 g/L to 110 g/L according our ROC results, naming it the modified ThroLy score (mThroLy). ROC curves indicated that the modified ThroLy score showed higher AUC than ThroLy score (0.710 vs. 0.695, Figure 2B). Then, we combined the mThroLy with D‐dimer in sequential testing and simultaneous testing (Figure 2B). Results showed that the modified ThroLy score combined with D‐dimer in simultaneous testing (simul‐mThroLy&D‐D) achieved the highest AUC value of 0.738, with a sensitivity of 76.2%, a specificity of 71.4%, and a Youden index of 47.6 (Figure 2C), indicating the optimized diagnostic efficiency.

Then, we collected a validation cohort comprising 98 NHL patients to assess the effectiveness of our combined method. Among these cases, 9 individuals (9.2%) developed VTE, with comparable age, gender, and lymphoma types observed between VTE‐positive and VTE‐negative patients (Table S4). According to the simul‐mThroLy&D‐D, we identified 21 individuals as high risk (mThroLy score> 3 or D‐dimer > 1345 μg/dL), with 6 experiencing VTE (6/21, 28.6%), while 3 patients in the low‐risk group also developed VTE (3/77, 3.9%). This discrepancy in VTE incidence rates between the high‐ and low‐risk groups was statistically significant (*p* = 0.002). In addition, simul‐mThroLy&D‐D yielded a sensitivity of 66.7%, specificity of 83.1%, Youden index of 49.8, PPV of 28.6%, NPV of 96.1%, with an AUC of 0.749 under the ROC curve (Figure 2D).

Taken together, this combined approach significantly improved VTE diagnosis in NHL patients, enhancing sensitivity without significant specificity loss.

### 3.5 Thrombomodulin and Thrombin–Antithrombin Complex Were Associated With VTE Development and Could Further Strengthen Diagnostic Efficiency

**FIGURE 3:**
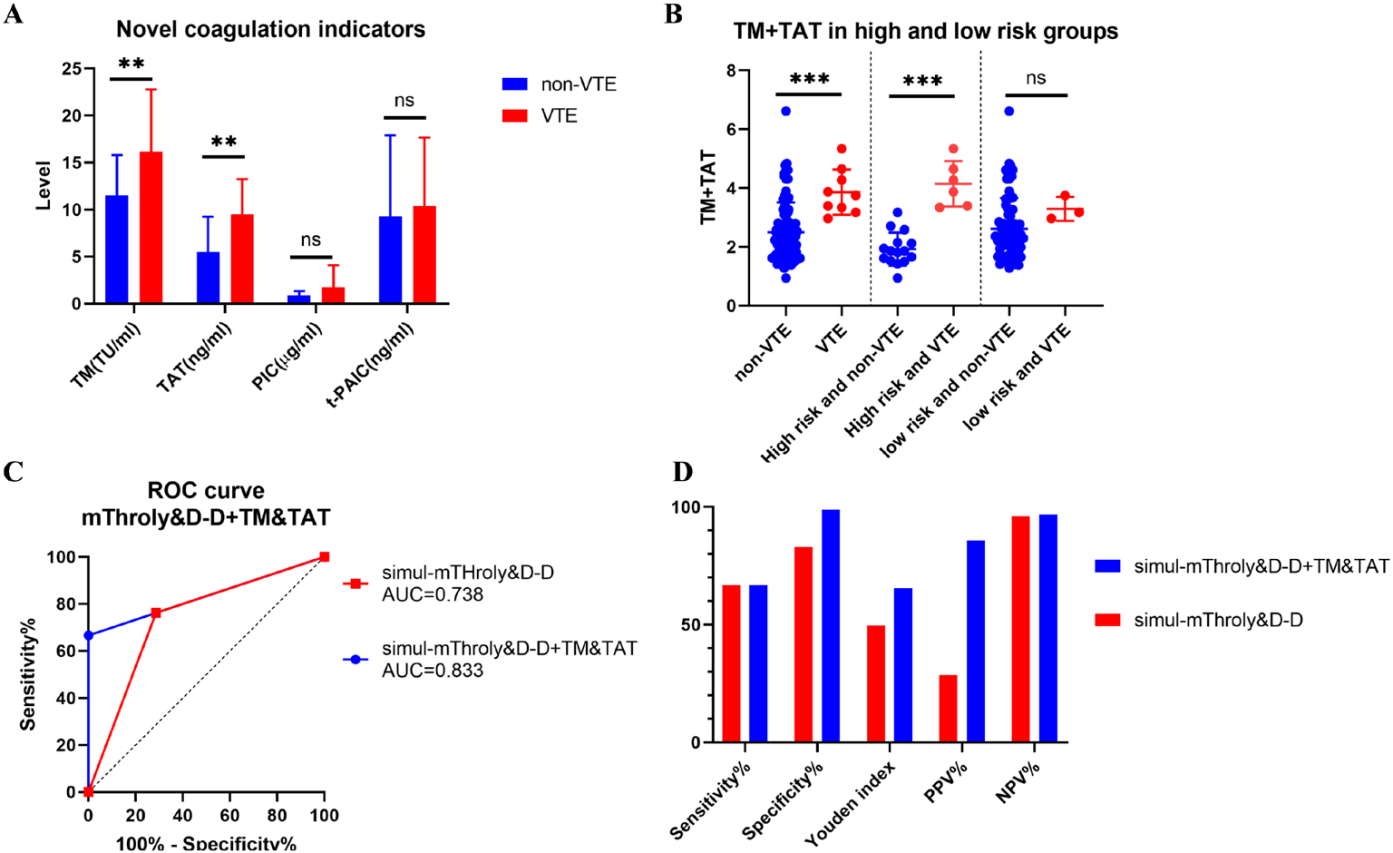
TM and TAT were associated with VTE development and could further strengthen diagnostic efficiency. (A) Levels of TM, TAT, PIC, and t‐PAIC in patients with and without VTE. (B) TM + TAT in patients with high‐ and low‐risk group based on simul‐mThroLy&D‐D. (C) ROC curves of simul‐mThroLy&D‐D + TM&TAT. (D) Comparison of the diagnostic efficiency of the above two risk models. PIC, plasminase–α2 plasminase inhibitor complex; TAT, thrombin–antithrombin complex; TM, thrombomodulin; t‐PAIC, tissue plasminogen activator/plasminogen activator inhibitor‐1 complex.

Furthermore, we examined the diagnostic efficacy of novel coagulation parameters, including TM, TAT, PIC, and t‐PAIC, for identifying VTE in our validation cohort. Results revealed that individuals who developed VTE exhibited significantly elevated levels of TM and TAT compared to those without VTE, while the levels of PIC and t‐PAIC remained comparable between the two groups (Figure 3A).

To comprehensively consider effects of TM and TAT on the development of VTE, we conducted logistic regression analysis and calculated the regression factor (*B* value) of TM and TAT. The combined effect of TM and TAT (TM + TAT) could be calculated by 0.125×TM + 0.194×TAT. Not surprisingly, TM + TAT was significantly higher in patients with VTE than those without VTE (*p* = 0.0002, Figure 3B). However, we found that only in the high‐risk group based on simul‐mThroLy&D‐D (mThroLy score > 3 or D‐dimer > 1345 μg/dL), rather than the low‐risk group, TM + TAT was significantly higher in patients with VTE than those without VTE (*p* < 0.0001 and *p* = 0.2698, Figure 3B).

Therefore, we considered whether the sequential testing of TM + TAT could enhance the diagnostic efficiency of simul‐mThroLy&D‐D (naming it simul‐mThroLy&D‐D + TM&TAT). We first determined the cutoff value of TM + TAT in high‐risk patients by ROC analysis, and it was 3.26. Then, we conducted simul‐mThroLy&D‐D. If it was identified as high risk (mThroLy score > 3 or D‐dimer > 1345 μg/dL), TM + TAT with the cutoff value of 3.26 was further assessed. Only patients with both high risk of simul‐mThroLy&D‐D and TM + TAT > 3.26 were identified as high risk of VTE. Based on above criteria, six patients were identified as high risk and all (100.0%) patients developed VTE, and the AUC value of simul‐mThroLy&D‐D + TM&TAT was 0.833 (Figure 3C). In addition, simul‐mThroLy&D‐D + TM&TAT yielded a sensitivity of 66.7%, specificity of 100.0%, Youden index of 66.7, PPV of 100.0%, and NPV of 96.7%, indicating a remarkable improvement of diagnostic efficiency (Figure 3D).

## 4 Discussion

Despite of an increasing trend in the incidence of VTE among hematologic malignancies, it remains an underestimated issue in NHL patients. In our cohort, we observed an incidence rate of 7.09% (30/423, including validation cohort). Mohren et al. [25] reported an incidence rate of 7.7% in lymphoma patients, and another study in Asian population showed a VTE rate of 7.9% in newly diagnosed lymphoma patients (8% in NHL and 6.7% in HL) [26]. Our results are consistent with these findings, although some studies reported VTE incidences exceeding 10%. In addition, our study revealed that VTE mostly occurred before chemotherapy or within 3 months of diagnosis and treatment, aligning with Caruso et al.'s [11] analysis of 18 studies, which found a 95% of occurrence rate during chemotherapy. It might be due to high tumor burden and the release of pro‐coagulant substances and inflammatory factors during chemotherapy, leading to endothelial damage and thrombosis formation [27]. In analyzing thrombus locations, we found a higher proportion of upper extremity vein thrombosis in NHL patients, which is consistent with previous research [9].

To investigate the risk factors of VTE in NHL patients, we comprehensively collected clinical characteristics. Our findings indicated that clinical Stage III/IV, mediastinal involvement, history of VTE, D‐dimer≥ 1345 μg/dL, and PLT≥ 298 × 10^9^ were independent risk factors for VTE, and Hb≥ 110 g/L was an independent protective factor for VTE. Advanced malignancy is closely associated with thrombosis formation. Our study showed that Stage III/IV NHL patients had a significantly higher VTE incidence (11.11%) compared to Stage I/II patients (2.33%). Additionally, 19.61% of patients with mediastinal involvement developed VTE, which is consistent with Borchmann et al. [28] reporting a higher rate of VTE in late‐stage lymphoma patients and Gartrell et al. [29] reporting the association between mediastinal mass and thrombosis.

The risk assessment of VTE is the precondition for prophylaxis, while the validation of present risk assessment models in NHL lymphoma patients is controversial and insufficient [18, 20]. We assessed the predictive value of the Khorana and ThroLy scores for VTE in NHL patients. The ThroLy score demonstrated superior predictive performance (AUC = 0.695) compared to the Khorana score (AUC = 0.639), though both had limited clinical predictive value. To improve the diagnostic efficiency of ThroLy score, we first compared a panel of hematological parameters between NHL patients with and without VTE, finding significant differences in NLR ratio, HB, PLT, and D‐dimer, which is consistent with previous studies [30, 31]. ROC curve analysis identified a D‐dimer threshold of 1345 ng/mL, achieving the highest diagnostic efficacy. In addition, the threshold of HB in ThroLy score was adjusted to 110 g/L. Result showed that the modified ThroLy score combined with D‐dimer in simultaneous testing achieved the highest AUC value of 0.738, with sensitivity of 76.2%, specificity of 71.4%. The result was further confirmed in the validation cohort, with a sensitivity of 66.7%, specificity of 83.1%, PPV of 28.6%, NPV of 96.1%, and an AUC of 0.749.

Although the simul‐ThroLy&D‐D improved VTE risk assessment, the PPV value was not ideal. Therefore, we further investigated roles of coagulation indicators TM, TAT, PIC, and t‐PAIC in VTE development. We found that patients with VTE exhibited significantly elevated levels of TM and TAT. The role of TM and TAT in coagulation and thrombosis has been widely demonstrated [32, 33], while limited evidences have been reported in NHL patients, and its predictive value in VTE remains unknown. Notably, we found that TM + TAT was exclusively higher in patients identified as high risk upon simul‐mThroLy&D‐D, and sequential testing of TM + TAT could remarkably enhance the diagnostic efficiency of simul‐mThroLy&D‐D, achieving an AUC value 0.833, a sensitivity of 66.7%, specificity of 100.0%, Youden index of 66.7, PPV of 100.0%, and NPV of 96.7%. Considering that the testing of TM and TAT has not been widely set up in most hospitals, the preliminary screening by simul‐ThroLy&D‐D, followed by TM + TAT testing if high risk is identified, could not only increase the diagnostic efficacy but also save healthcare costs.

Preventing thrombotic complications in hematological malignancies remains a controversial issue. The American Society of Clinical Oncology (ASCO) and the International Association for Cancer and Thrombosis (IACT) do not recommend routine primary thromboprophylaxis for cancer patients undergoing treatment. However, they suggest implementing VTE prevention for cancer patients at high risk of thrombosis [34, 35]. In clinical practice, the combined simul‐ThroLy&D‐D plus TM + TAT model, with a PPV of 100%, would effectively guide our clinical decisions. Low molecular weight heparin (LMWH) is generally recommended for the prevention of thromboembolism in patients with hematological malignancies [36, 37]. Additionally, the AVERT and CASSINI randomized trials examined the use of direct oral anticoagulants (DOACs) as primary prophylaxis for high‐risk cancer patients, demonstrating DOACs may lower the incidence of VTE in selected populations, but further research is needed to identify specific groups that can clearly benefit from thromboprophylaxis [38, 39].

## 5 Conclusion

In conclusion, VTE is a significant complication in NHL patients. Our study identified an incidence rate of 7.09% and highlighted several independent risk factors. The modified ThroLy score simultaneously combined with D‐dimer levels offers a more effective prediction method for VTE risk in NHL patients, and further inclusion of coagulation markers TM and TAT could enhance predictive accuracy, potentially optimizing VTE prophylaxis and reducing healthcare costs.

## Author Contributions

**Wen Li:** data curation (equal), formal analysis (equal), investigation (equal), methodology (equal). **Rui Liu:** methodology (equal), validation (equal), visualization (equal), writing – original draft (equal). **Ying Shen:** formal analysis (equal), methodology (equal), validation (equal). **GongZhizi Gao:** formal analysis (equal), writing – review and editing (equal). **Rui Yang:** formal analysis (equal), writing – review and editing (equal). **Yiwen Wang:** formal analysis (equal), writing – review and editing (equal). **Ruoyu Yang:** formal analysis (equal), writing – review and editing (equal). **Zujie Lin:** formal analysis (equal), writing – review and editing (equal). **Ruijun Dong:** formal analysis (equal), writing – review and editing (equal). **Wanhong Zhao:** writing – review and editing (equal). **Aili He:** conceptualization (equal), methodology (equal), writing – review and editing (equal). **Ju Bai:** conceptualization (equal), methodology (equal), writing – review and editing (equal).

## Ethics Statement

This study was conducted in accordance with the Declaration of Helsinki and was approved by the institutional independent ethics committee of The Second Affiliated Hospital of Xi'an Jiaotong University (No. 2016228).

## Consent

All patients gave written informed consent.

## Conflicts of Interest

The authors declare no conflicts of interest.

## Permission to Reproduce Material From Other Sources

The authors have nothing to report.

## Supporting information


Data S1.

## Data Availability

Data sharing not applicable to this article as no datasets were generated or analyzed during the current study.
