# Supplementary material for: Incidence, Risk Factors, and Modified Risk Assessment Model of Venous Thromboembolism in Non‐Hodgkin Lymphoma Patients
Source: Cancer Med. 2024 Dec 23;13(24):e70510. doi: 10.1002/cam4.70510 (PMC11664237; doi:10.1002/cam4.70510)
Supplement: Supplementary file 1 — Data S1. [file CAM4-13-e70510-s001.docx]

**Supplementary Table 1. Khorana score for VTE**

| Patient characteristic | Risk score |
| --- | --- |
| Stomach, pancreas cancer | 2 |
| Lung, lymphoma, gynecologic, bladder, testicular cancer | 1 |
| Prechemotherapy platelet count ≥350×10^9/^L | 1 |
| Hemoglobin <100 g/L | 1 |
| Prechemotherapy leukocyte count >11×10^9^/L | 1 |
| BMI ≥35 kg/m^2^ | 1 |

High risk ≥3；Intermediate risk：1-2；Low risk：0

BMI: body mass index; VTE: venous thromboembolic events

**Supplementary Table 2. ThroLy score for VTE**

| Patient characteristic | Risk score |
| --- | --- |
| Previous VTE/AMI/stroke | 2 |
| Reduced mobility (ECOG 2-4) | 1 |
| Obesity (BMI >30 kg/m^2^) | 2 |
| Extranodal localization | 1 |
| Mediastinal involvement | 2 |
| Neutrophils < 1×10^9^/L | 1 |
| Hemoglobin level <100 g/L | 1 |

High risk >3；Intermediate risk：2-3；Low risk：0-1

AMI: acute myocardial infarction; BMI: body mass index; VTE: venous thromboembolic events

**Supplementary Table 3. General Characteristics of 325 NHL patients**

| **General Characteristics** | | **Cases (%)** |
| --- | --- | --- |
| Gender | Male | 168 (51.7) |
|  | Female | 157 (48.3) |
| Age | >60 years | 171 (52.6) |
|  | ≤60 years | 154 (47.4) |
| NHL Type | Diffuse Large B-cell Lymphoma | 159 (48.9) |
|  | NK/T-cell Lymphoma | 43 (13.2) |
|  | Peripheral T-cell Lymphoma | 33 (10.2) |
|  | Follicular Lymphoma | 18 (5.4) |
|  | Mantle Cell Lymphoma | 14 (4.3) |
|  | Small Lymphocytic Lymphoma | 10 (3.1) |
|  | Anaplastic Large Cell Lymphoma | 10 (3.1) |
|  | Splenic Marginal Zone Lymphoma | 9 (2.8) |
|  | Angioimmunoblastic T-cell Lymphoma | 9 (2.8) |
|  | Mucosa-associated Lymphoid Tissue Lymphoma | 8 (2.5) |
|  | T-cell Prolymphocytic Leukemia | 7 (2.2) |
|  | Lymphoplasmacytic Lymphoma | 5 (1.5) |
| Ann Arbor Stage | Stage I | 69 (21.2) |
|  | Stage II | 103 (31.7) |
|  | Stage III | 77 (23.7) |
|  | Stage IV | 76 (23.4) |
| ECOG | 0 | 41 (12.6) |
|  | 1 | 86 (26.5) |
|  | 2 | 144 (44.3) |
|  | 3 | 3 (0.9) |
|  | 4 | 10 (3.1) |
|  | 5 | 41 (12.6) |

**Supplementary Table 4. General Characteristics of 98 NHL patients in validation cohort**

| **Risk Factor** | **Non-VTE (%)** | **VTE (%)** | **P-value** |
| --- | --- | --- | --- |
| **Age** | 58.66±13.279 | 59±8.216 | 0.941 |
| **Gender** |  |  | 0.293 |
| Male | 49 (87.5) | 7 (12.5) |  |
| Female | 40 (95.2) | 2 (4.8) |  |
| **NHL Type** |  |  | 0.698 |
| Diffuse Large B-Cell Lymphoma | 46 (92) | 4 (8) |  |
| Peripheral T-Cell Lymphoma | 8 (80) | 2 (20) |  |
| Mantle Cell Lymphoma | 8 (100) | 0 (0) |  |
| Mucosa-Associated Lymphoid Tissue Lymphoma | 6 (85.7) | 1 (14.3) |  |
| Mantle Cell Lymphoma | 5 (83.3) | 1 (16.7) |  |
| Follicular Lymphoma | 5 (100) | 0 (0) |  |
| NK/T-Cell Lymphoma | 4 (100) | 0 (0) |  |
| Large B-Cell Lymphoma | 3 (75) | 1 (25) |  |
| Angioimmunoblastic T-Cell Lymphoma | 3 (100) | 0 (0) |  |
| Lymphoplasmacytic Lymphoma | 1 (100) | 0 (0) |  |
